# Exchange of Quantitative Computed Tomography Assessed Body Composition Data Using Fast Healthcare Interoperability Resources as a Necessary Step Toward Interoperable Integration of Opportunistic Screening Into Clinical Practice: Methodological Development Study

**DOI:** 10.2196/68750

**Published:** 2025-05-21

**Authors:** Yutong Wen, Vin Yeang Choo, Jan Horst Eil, Sylvia Thun, Daniel Pinto dos Santos, Johannes Kast, Stefan Sigle, Hans-Ulrich Prokosch, Diana Lizzhaid Ovelgönne, Katarzyna Borys, Judith Kohnke, Kamyar Arzideh, Philipp Winnekens, Giulia Baldini, Cynthia Sabrina Schmidt, Johannes Haubold, Felix Nensa, Obioma Pelka, René Hosch

**Affiliations:** 1 Data Integration Center Central IT Department University Hospital Essen Essen Germany; 2 Institute for Artificial Intelligence in Medicine (IKIM) University Hospital Essen Essen Germany; 3 Digital Medicine and Interoperability Berlin Institute of Health (BIH) at Charité - University Hospital Berlin Berlin Germany; 4 University Medical Center Mainz Mainz Germany; 5 Mint Medical GmbH (a Brainlab company) Heidelberg Germany; 6 Gematik Expert Group Gematik GmbH Berlin Germany; 7 MOLIT Institut für personalisierte Medizin gGmbH Heilbronn Germany; 8 Institut für Medizininformatik, Biometrie und Epidemiologie Lehrstuhl für Medizinische Informatik Friedrich-Alexander-Universität Erlangen-Nürnberg Erlangen Germany; 9 Siemens Healthineers AG Forchheim Germany; 10 Institute of Diagnostic and Interventional Radiology and Neuroradiology University Hospital Essen Essen Germany; 11 Institute for Transfusion Medicine University Hospital Essen Essen Germany; 12 Center of Sleep and Telemedicine University Hospital Essen-Ruhrlandklinik Essen Germany

**Keywords:** HL7 fast healthcare interoperability resources, HL7 FHIR profiling, health information interoperability, opportunistic screening in radiology, body composition analysis

## Abstract

**Background:**

Fast Healthcare Interoperability Resources (FHIR) is a widely used standard for storing and exchanging health care data. At the same time, image-based artificial intelligence (AI) models for quantifying relevant body structures and organs from routine computed tomography (CT)/magnetic resonance imaging scans have emerged. The missing link, simultaneously a needed step in advancing personalized medicine, is the incorporation of measurements delivered by AI models into an interoperable and standardized format. Incorporating image-based measurements and biomarkers into FHIR profiles can standardize data exchange, enabling timely, personalized treatment decisions and improving the precision and efficiency of patient care.

**Objective:**

This study aims to present the synergistic incorporation of CT-derived body organ and composition measurements with FHIR, delineating an initial paradigm for storing image-based biomarkers.

**Methods:**

This study integrated the results of the Body and Organ Analysis (BOA) model into FHIR profiles to enhance the interoperability of image-based biomarkers in radiology. The BOA model was selected as an exemplary AI model due to its ability to provide detailed body composition and organ measurements from CT scans. The FHIR profiles were developed based on 2 primary observation types: Body Composition Analysis (BCA Observation) for quantitative body composition metrics and Body Structure Observation for organ measurements. These profiles were structured to interoperate with a specially designed Diagnostic Report profile, which references the associated Imaging Study, ensuring a standardized linkage between image data and derived biomarkers. To ensure interoperability, all labels were mapped to SNOMED CT (Systematized Nomenclature of Medicine – Clinical Terms) or RadLex terminologies using specific value sets. The profiles were developed using FHIR Shorthand (FSH) and SUSHI, enabling efficient definition and implementation guide generation, ensuring consistency and maintainability.

**Results:**

In this study, 4 BOA profiles, namely, Body Composition Analysis Observation, Body Structure Volume Observation, Diagnostic Report, and Imaging Study, have been presented. These FHIR profiles, which cover 104 anatomical landmarks, 8 body regions, and 8 tissues, enable the interoperable usage of the results of AI segmentation models, providing a direct link between image studies, series, and measurements.

**Conclusions:**

The BOA profiles provide a foundational framework for integrating AI-derived imaging biomarkers into FHIR, bridging the gap between advanced imaging analytics and standardized health care data exchange. By enabling structured, interoperable representation of body composition and organ measurements, these profiles facilitate seamless integration into clinical and research workflows, supporting improved data accessibility and interoperability. Their adaptability allows for extension to other imaging modalities and AI models, fostering a more standardized and scalable approach to using imaging biomarkers in precision medicine. This work represents a step toward enhancing the integration of AI-driven insights into digital health ecosystems, ultimately contributing to more data-driven, personalized, and efficient patient care.

## Introduction

The modern health care landscape is filled with diverse data sources, ranging from electronic medical records to wearable devices, containing a wide range of patient information that promises to transform health care. A key challenge holding back this transformative potential is the issue of health data interoperability. Data interoperability, the ability of disparate information technology systems and software applications to communicate, exchange, and interpret shared data, is at the heart of creating a seamlessly integrated health care ecosystem [1]. The Fast Healthcare Interoperability Resources (FHIR) standard [2] is at the forefront of addressing this interoperability challenge. Developed by Health Level Seven International (HL7), FHIR provides a robust framework for exchanging, integrating, sharing, and retrieving electronic health information.

At the same time, the emergence of artificial intelligence (AI) in health care offers unprecedented opportunities for improved patient outcomes, predictive analytics, and personalized medicine. However, data extracted by AI algorithms often exist in isolated environments, making it difficult to integrate with existing health care data systems [3]. Standardizing AI-generated data to be FHIR conformant ensures that this valuable information is standardized and interoperable. This interoperable framework facilitates the seamless merging of AI insights with existing patient records, increasing the clinical utility of AI-derived data.

Advancements in AI and imaging modalities such as computed tomography (CT) have revolutionized body composition analysis by enabling the automated extraction of clinically relevant biomarkers [4-6]. These biomarkers, quantifying skeletal muscle, adipose tissue, bone mineral density, and organ volumes, offer a detailed physiological portrait of patients that directly informs clinical decision-making. Across a wide range of medical disciplines, including oncology, cardiology, surgery, and metabolic care, these data support risk stratification, guide treatment planning, predict therapeutic response, and improve prognostic accuracy.

For example, skeletal muscle mass and fat distribution are increasingly used to predict chemotherapy toxicity and survival in patients with cancer, directly influencing treatment regimens [7-9]. In surgical care, preoperative assessment of muscle and fat composition enhances perioperative risk evaluation and postoperative management [10,11]. In cardiology, quantifying visceral fat from routine CT scans enables more accurate cardiovascular risk assessment than traditional biomarkers alone [12,13]. AI-based bone mineral density estimation allows for opportunistic osteoporosis screening, promoting earlier interventions to prevent fractures [14,15]. Therefore, these imaging-derived features could not only inform diagnostic and prognostic assessments but also facilitate longitudinal monitoring through the integration of multiple clinical parameters across time points [16-18].

Despite these benefits, the integration of AI-derived biomarkers into clinical workflows remains limited due to the absence of standardized, interoperable formats for health data exchange. This disconnect impedes the translation of imaging-based insights into actionable information within electronic health records and clinical decision support systems. The FHIR standard offers a promising solution, enabling structured, computable representation of clinical data. However, existing FHIR profiles lack the necessary specificity to accommodate CT-based body composition biomarkers, limiting their accessibility and utility in real-world care settings. Central to this approach is the Body and Organ Analysis (BOA) model [19,20], a deep-learning segmentation tool that quantifies tissues, organs, and bone mineral density from CT scans. In this study, BOA is used as an example model that enables the generation of biomarkers that can be mapped to FHIR resources, making them potentially widely usable within digital health platforms.

Therefore, this study aims to develop and demonstrate FHIR profiles for the standardized representation and exchange of CT-derived imaging biomarkers, using the BOA model as a use case to bridge the integration gap between AI-driven body composition analytics and digital health systems.

## Methods

### Background

FHIR [21] is an open-standard accommodating semantic representation for a diverse spectrum of health care data, fostering interoperability. For instance, laboratory data can be encapsulated within the Observation resource, capturing parameters like blood glucose levels or white blood cell counts. Imaging studies, which include radiology reports and associated images such as x-rays or magnetic resonance imaging (MRI), find their place in the Imaging Study and Diagnostic Report resources. The diagnostic or surgical procedures can be documented within the Procedure resource. Furthermore, pivotal patient diagnoses in treatment plans and medical histories are housed within the Condition resource. Through a flexible information model, FHIR ensures that varied health care data types, from laboratory results to imaging studies and clinical information, are seamlessly integrated and easily accessible within a uniform, standardized, and relational data format.

FHIR offers a structured framework with predefined resources designed to address common health care data needs, adhering to the 80/20 rule, focusing on 20% of requirements that satisfy 80% of interoperability demands [22]. However, due to the complexity and evolving nature of health care, not all data fits perfectly into standard FHIR resources. Medical technologies, AI, research findings, and unique patient care needs often require customization. To accommodate these specific cases, FHIR supports the creation of “profiles,” which are custom-designed schemas that adapt and extend base FHIR resources. These profiles ensure that even niche data types maintain interoperability and standardization. All the profiles in this study were created using FHIR Shorthand (FSH) [23] in alignment with the FHIR Release 4 version.

### The BOA Model

**Figure 1:**
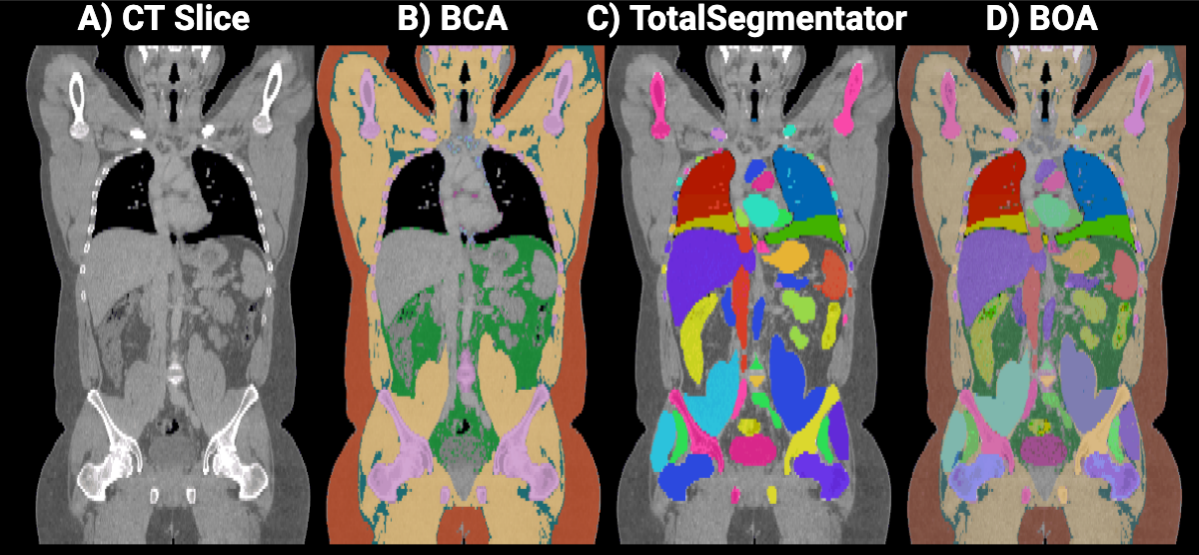
Exemplary visualization of the voxel body coverage on the middle coronal section of volume (A) based on the segmentation masks of Body Composition Analysis (B), TotalSegmentator (C), and BOA algorithms (D). BCA: Body Composition Analysis; BOA: Body and Organ Analysis; CT: computed tomography. Created in BioRender [26].

BOA [19,20] is a CT scan segmentation algorithm that can analyze medical images and identify various anatomical structures and tissue types, including bones, muscles, and organs. The BOA merges 2 different segmentation algorithms: Body Composition Analysis (BCA) trained on nnU-Net [24] and the open-source TotalSegmentator [25], providing an all-inclusive segmentation with a focus on workflow integration [19] (Figure 1).

The BCA segments body compositions across different anatomical regions in CT images and provides critical measurements, including muscle volume, bone density, volume of subcutaneous adipose tissue and visceral adipose tissue, intramuscular adipose tissue volume, as well as the Hounsfield unit density of these tissues. These body composition measurements play a relevant role in current research as a predictive factor for various diseases [5,7,19].

Organ measurements are essential to medical diagnostics and monitoring [27]. The advent of sophisticated imaging modalities, such as CT and MRI, has significantly augmented this assessment process. Such evaluations, which focus on size, volume, and density, facilitate detailed insights into organ function and structure [28]. They enhance clinical decision-making in various medical fields, including oncology [29,30], leading to more efficient and tailored health care delivery.

By combining BCA with the TotalSegmentator algorithm, BOA provides a rich and detailed set of features, including precise measurements and biomarkers, and computes a precise assessment of patients’ body composition [19]. Furthermore, tools like the BOA could be used during clinical routine CT examinations, supporting opportunistic screening [31] with additional and incidental information that exceeds the original examination.

### Opportunistic Screening in Radiology

**Figure 2:**
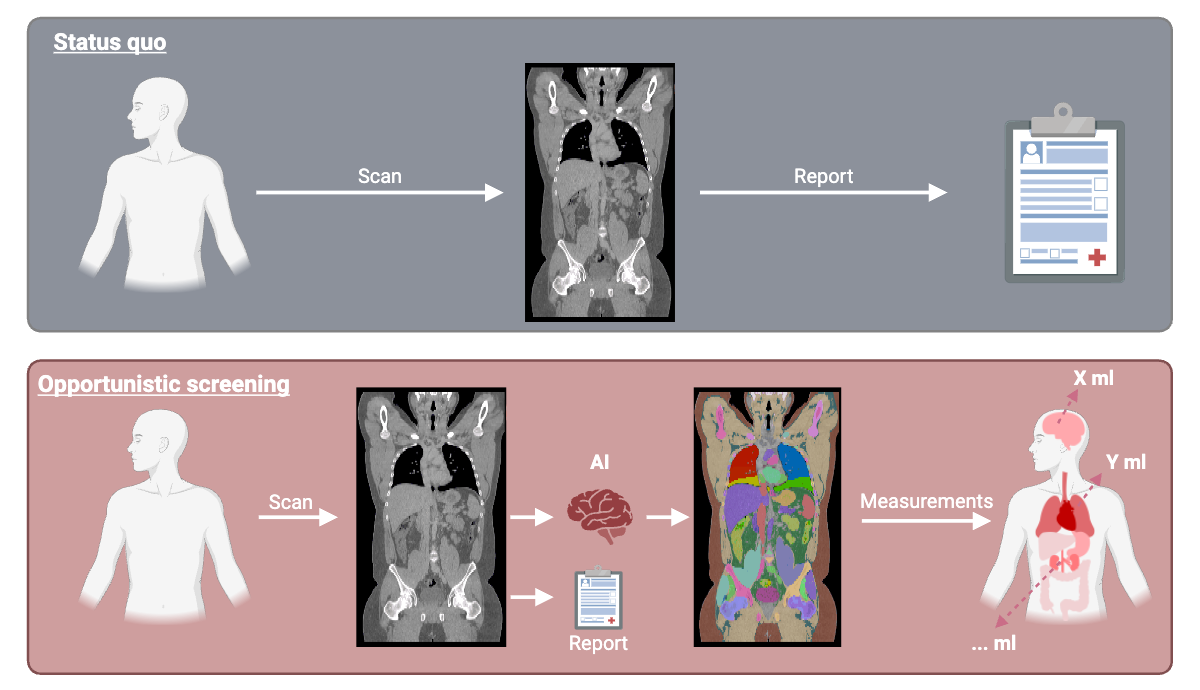
Visualization of opportunistic screening with Body and Organ Analysis. The upper image block (gray) represents the current routine, where patients undergo computed tomography scans and receive a report. The lower image block (pink) illustrates artificial intelligence–based opportunistic screening, quantitatively assessing tissues and image-based biomarkers alongside the standard report. AI: artificial intelligence. Created in BioRender [34].

Opportunistic screening in radiology refers to using routine radiological scans, such as CT scans, MRIs, and X-rays, to detect or quantify features that are present in the image but unrelated to the clinical indication [32,33] or too complicated to extract for radiologists (eg, muscle volume in the abdominal cavity). This approach enables a quantitative approach for patient comparison and enables the generation of image-based biomarkers using the full image instead of only using it for report generation (Figure 2).

The quantitative assessment of body composition (eg, bone, fat, and muscle tissue) and other related biomarkers, such as vascular calcification or organ volume and density [33,35], can be valuable for risk stratification and comparison [33]. Many AI models, such as the BOA [19], can rapidly quantify volumes and precisely provide biomarkers, significantly enhancing the information depth based on routine imaging examinations. Furthermore, these automated tools can process large data volumes with high accuracy without adding procedural burdens to radiologists’ workload.

### Opportunistic Screening Workflow

**Figure 3:**
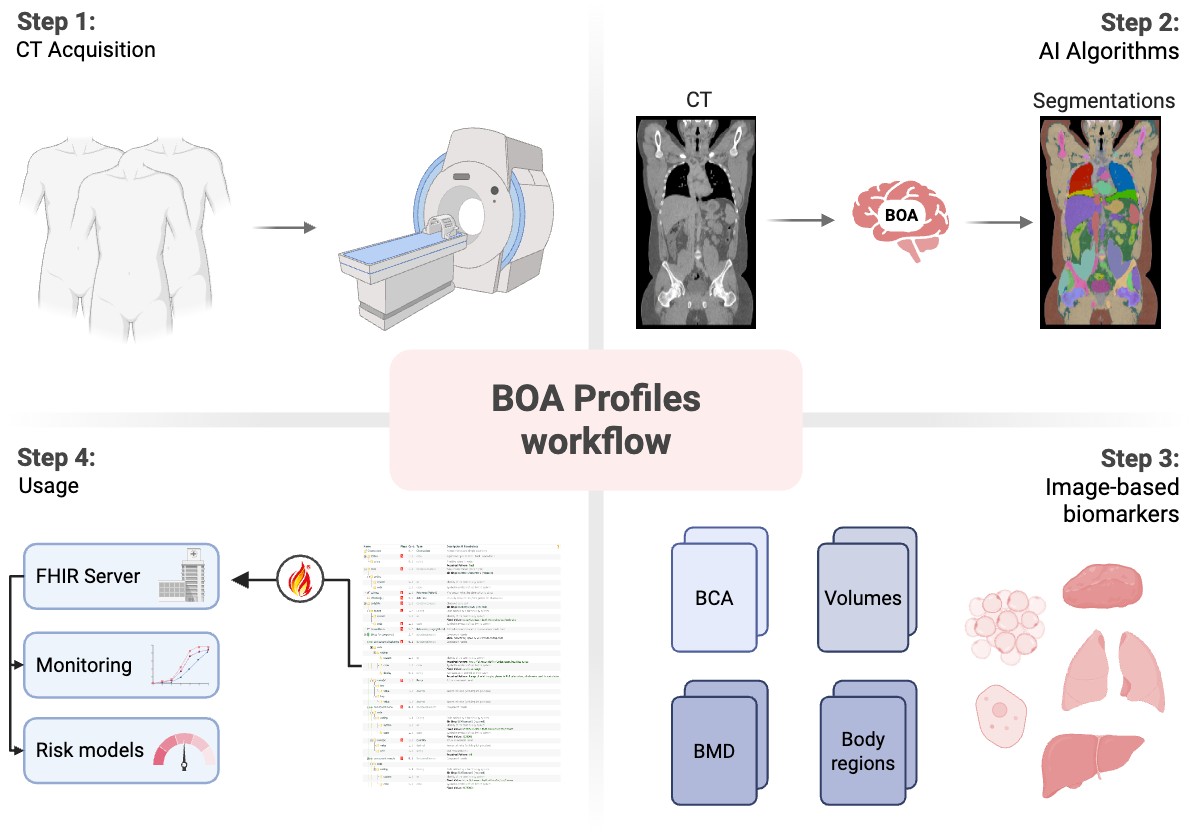
The entire opportunistic screening process workflow, including Fast Healthcare Interoperability Resources and usage. BOA: Body and Organ Analysis; BCA: Body Composition Analysis; BMD: Bone Mineral Density; FHIR: Fast Healthcare Interoperability Resources. Created in BioRender [36].

An example of data flow for the opportunistic screening, including the FHIR profiles, is shown in Figure 3. The initial step entails the patient undergoing a radiological scan such as CT, which is subsequently processed by AI models such as BOA.

In this phase, the different body structures are segmented, and various biomarkers are generated by detailed evaluation of the anatomical structures and tissues. After completion of the AI processing, the acquired information is represented in conformance to the BOA FHIR profiles and persists on the FHIR server, facilitating their usage for downstream analysis, including use cases such as risk stratification of patients to predict mortality or longitudinal comparison of patients’ physical composition to monitor changes for personalized care.

### FHIR Profile Creation Process

The development of the BOA FHIR profiles was guided by the need for a structured, standardized, and interoperable representation of AI-derived imaging biomarkers. The outputs of the BOA model, which include body composition metrics (eg, muscle and fat quantifications) and organ volumetrics (eg, liver and spleen volumes), served as the blueprint for 2 key FHIR Observation profiles: BOA BCA Observation and BOA Body Structure Volume Observation. The BCA Observation profile was designed to store AI-derived body composition measurements, while the Body Structure Volume Observation profile captures organ volumetrics, structuring each anatomical region as a discrete component containing a CodeableConcept (structure name) and a Quantity (volume in mL). To ensure interoperability across health care systems, SNOMED CT (Systematized Nomenclature of Medicine – Clinical Terms) and RadLex codings were mapped to all labels within the profiles. SNOMED CT was used for body sites (eg, “abdominal cavity” and “thoracic cavity”), while RadLex codings were applied for imaging-related terms, enhancing radiology-specific integration. Additionally, custom value sets such as BCAMeasurementsVS (for measurement types), and BCABodySiteVS (for anatomical locations) were developed to standardize terminology across different implementations.

To align with clinical imaging workflows, a BOA Diagnostic Report profile was created as a central aggregator, connecting all AI-derived observations. It references the DICOM (Digital Imaging and Communications in Medicine) Imaging Study Unique Identifier, ensuring a direct association between AI-generated biomarkers and imaging data. The BOA Imaging Study profile was designed to reference only the specific series used for AI inference rather than the entire imaging study, improving traceability and reproducibility. Furthermore, the Read Procedure profile designed by the Medical Informatics Initiative (MII) [37] was integrated to document metadata related to the AI model parameters and computational settings, providing essential context for clinicians and researchers interpreting the results. The profiling process was implemented using FSH and compiled with SUSHI [38], enabling a concise and structured definition of the BOA profiles. This approach facilitated rapid iteration and validation of the profiles while maintaining consistency across different implementations. By leveraging SUSHI, we automated the transformation of the FSH and facilitated the generation of FHIR Implementation Guides, ensuring that all constraints, extensions, and terminology bindings were systematically documented and versioned. This methodology streamlined the profiling workflow and improved maintainability as BOA evolves to support additional AI-driven imaging biomarkers.

### Ethical Considerations

This study did not require ethical approval as it did not involve human participants, patient data, medical records, or direct observation of public behavior. The research focused solely on the development of FHIR profiles based on AI model definitions without the use of identifiable or sensitive human data. As no personal health information or secondary data involving human participants was used, the study falls outside the scope of human participants’ research, requiring ethical review in accordance with institutional and regulatory guidelines.

## Results

### Overview

**Figure 4:**
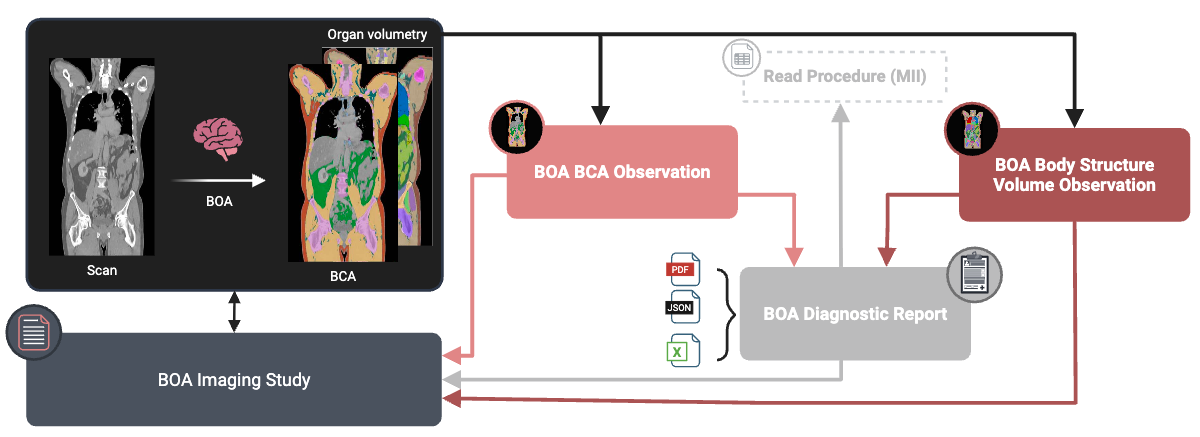
Overview of the Fast Healthcare Interoperability Resources profiles. The 4 Body and Organ Analysis (BOA) Fast Healthcare Interoperability Resources profiles (BOA BCA Observation, BOA Body Structure Volume Observation, BOA Diagnostic Report, and BOA Imaging Study) and Read Procedure (Medical Informatics Initiative) are closely interlinked and play a central role in clinical documentation and diagnostics. BOA: Body and Organ Analysis; BCA: Body Composition Analysis; MII: Medical Informatics Initiative. Created in BioRender [39].

The development process resulted in 4 BOA profiles based on the FHIR resources Observation, Diagnostic Report, and Imaging Study. The overall link between those resources is shown in Figure 4.

The BOA BCA Observation profile accommodates BCA measurements from BOA, including terminology coding of the body composition. The second Observation profile, BOA Body Structure Volume, focuses on recording and documenting the volume of body structures. The BOA Diagnostic Report profile is a comprehensive summary of the quantifications in different formats (JSON, PDF report, and Microsoft Excel) derived by the BOA, and these results are linked to BOA BCA and Body Structure Volume Observation. The Read Procedure profile is used to record diagnostic procedures, and the report on the procedure is linked to the Diagnostic Report. The BOA Imaging Study profile documents the specific series used for the AI algorithm from a respective Imaging Study. It records the imaging details, including the study series and their characteristics, which is the basis for the observations and diagnostic reports recorded in the other profiles. A detailed overview of the individual profiles is presented as follows.

### BOA BCA Observation

**Figure 5:**
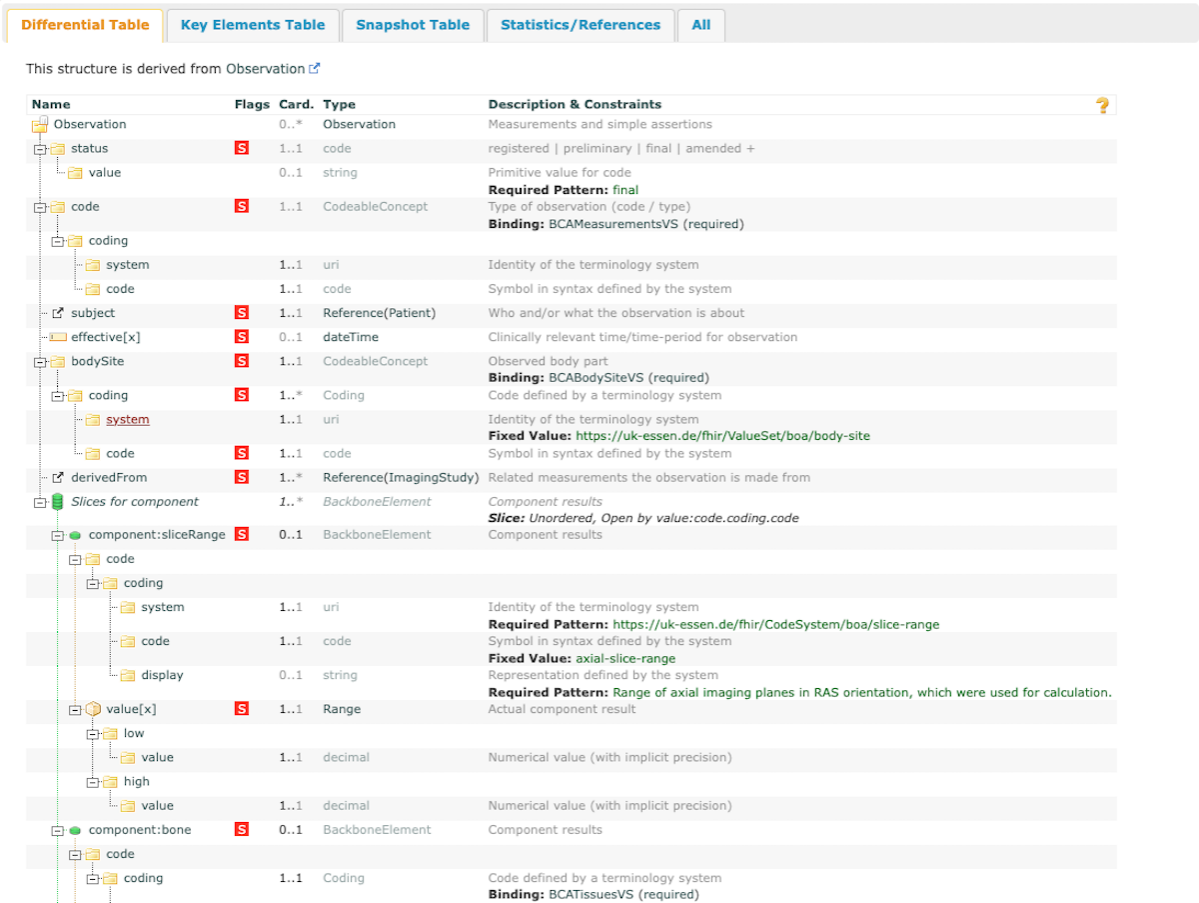
Visualization of Body and Organ Analysis (BOA) Body Composition Analysis (BCA) Observation profile. A comprehensive overview of the Fast Healthcare Interoperability Resources profile BOA BCA Observation, with a particular emphasis on the aspects related to observation status, “bodySite,” and the imaging-derived components.

The first FHIR profile BOA BCA Observation is based on the Observation Resource, which documents medical measurements and observations (Figure 5).

In addition to the standard elements present in the FHIR profile, such as “subject” and “status,” this profile changes the value set binding. For example, the “code” element is associated with the “BCAMeasurementsVS” value set (Figure S2 in Multimedia Appendix 1), which includes the code of measurements with or without extremities. There is a fixed binding to the “BCABodySiteVS” value set in this profile (Figure S2 in Multimedia Appendix 1). The value set includes the code of “abdominal cavity,” “thoracic cavity,” “mediastinum,” and “pericardium” defined in “bodySite” and SNOMED CT. Furthermore, the Observation resource comprises more intricate structures in “component” elements, further specifying diverse observation aspects. For example, the results of a specific slice range can be delineated using the “component:sliceRange” component. These components offer the possibility of documenting specific details from the imaging examination in a structured and standardized way.

### BOA Body Structure Volume Observation

**Figure 6:**
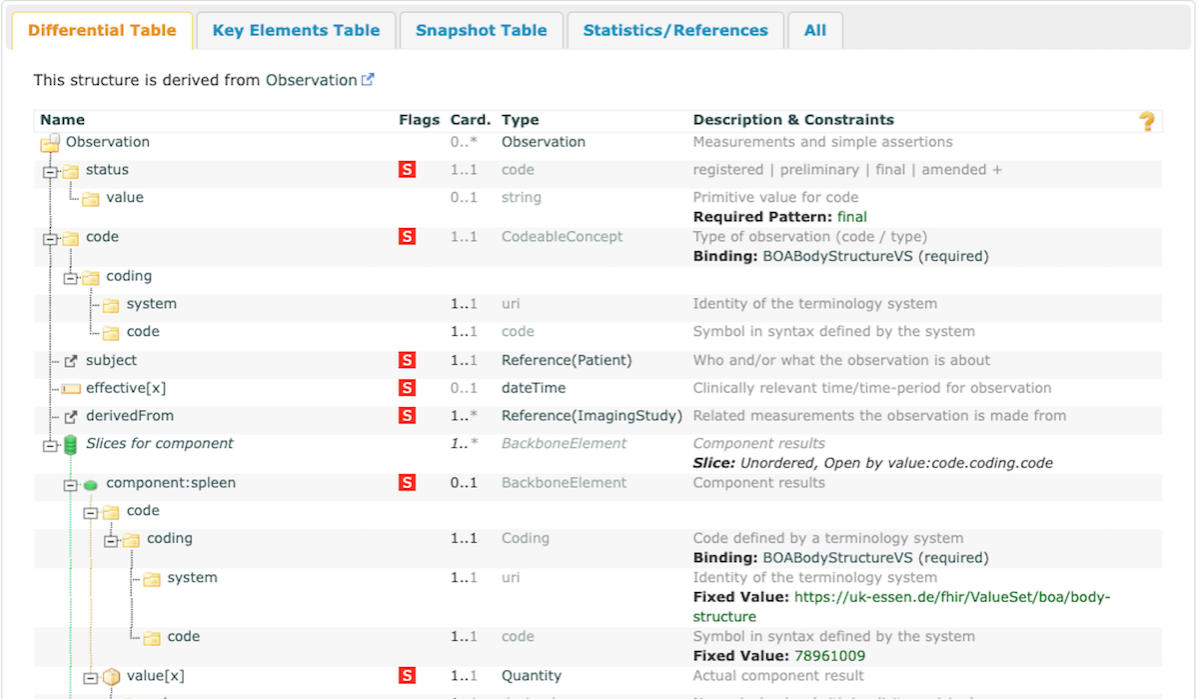
Schematic representation of the Fast Healthcare Interoperability Resources Body and Organ Analysis (BOA) Body Structure Volume Observation profile, demonstrating the use of components to document anatomical regions and volumes.

The BOA Body Structure Volume Observation depicted in Figure 6 was developed based on the Observation resource and documents particular observations about anatomic regions. The structure of this profile is similar to that of BCA Observation, and each of these observations is structured as a discrete component. The body structures are detailed within the components, each identified by anatomical landmarks, for example, “component: spleen.” These components contain a mandatory code (“CodeableConcept”), which identifies the respective structure, and a numerical value (“Quantity”), which specifies the volume of the structure in milliliters. This structure guarantees that all observations are accurately documented and explicitly associated with the corresponding imaging examinations, thus ensuring consistency and precision in clinical documentation.

### BOA Diagnostic Report

**Figure 7:**
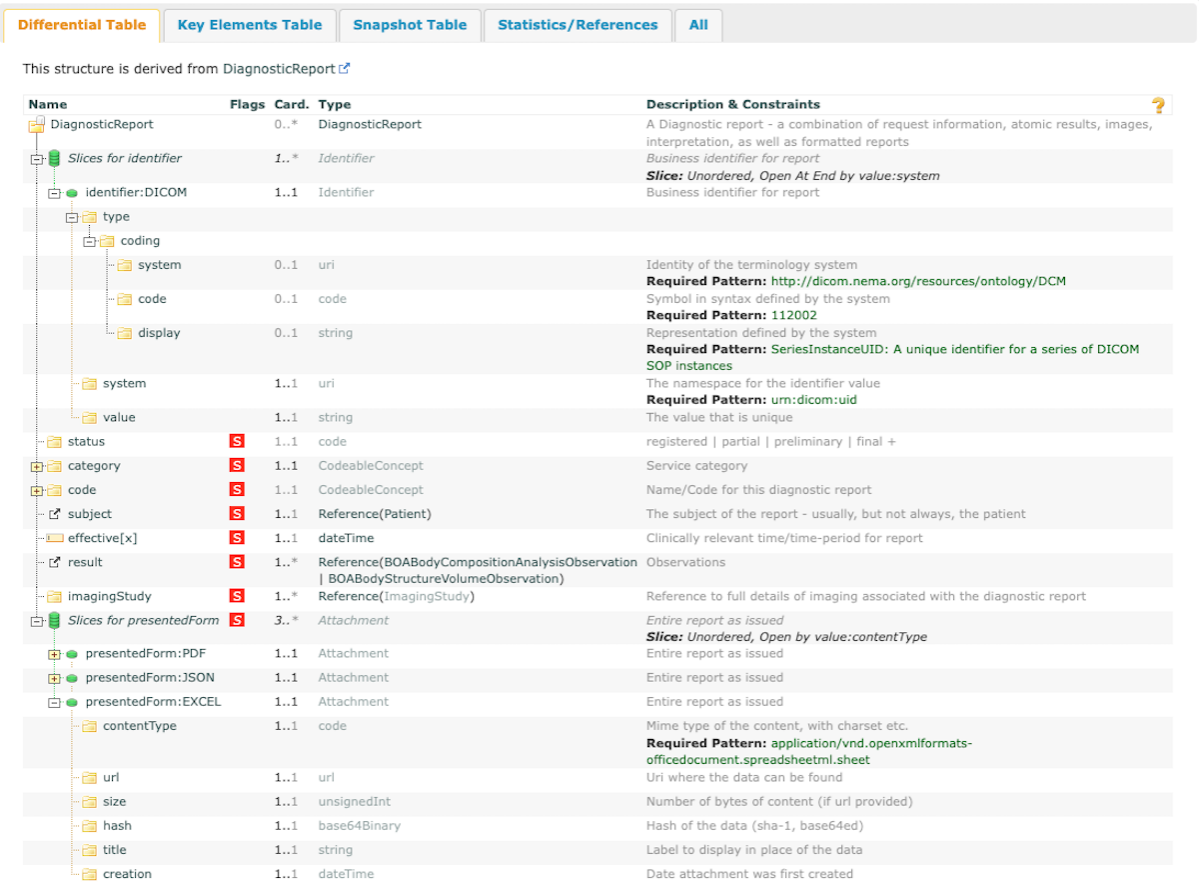
Visualization of Body and Organ Analysis (BOA) Diagnostic Report profile. Abbreviated layout of the core elements in the Fast Healthcare Interoperability Resources profile for BOA Diagnostic Report, focusing on essential components such as DICOM (Digital Imaging and Communications in Medicine) identifiers and report presentation formats.

The BOA Diagnostic Report FHIR profile has been developed to document diagnostic reports, and an excerpt of the profile is visualized in Figure 7. The “identifier: DICOM” provides a unique DICOM identifier, defined according to the “urn:dicom:uid” pattern. The FHIR profile refers to the presentation of diagnostic reports, explicitly focusing on the diverse formats in which these reports can be accessed. The structure contains references to the Imaging Study resource, results from the BOA algorithm referring to other BOA Observation profiles, and various file formats labeled “presentedForm,” such as PDF, JSON, or Microsoft Excel, depending on the user’s needs.

The Read Procedure profile, implemented by the German “Medical Informatics Initiative,” describes the diagnostic procedure and is linked to the BOA Diagnostic Report in this study. The metadata in the procedure can specify detailed information about both the AI and the computational parameters, which can provide clinicians with more accurate context to better classify the results.

### BOA Imaging Study

**Figure 8:**
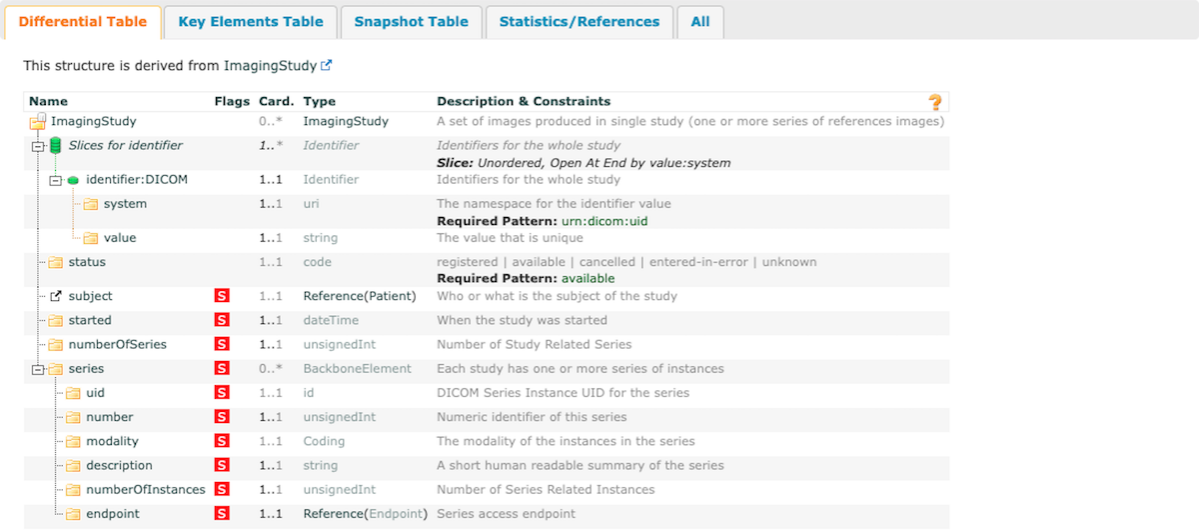
Visualization of Body and Organ Analysis (BOA) Imaging Study profile. A structured overview of the Fast Healthcare Interoperability Resources profile for BOA Imaging Study, outlining the key elements for managing image series and associated metadata, like series information, initiation date, and DICOM (Digital Imaging and Communications in Medicine) identifiers.

The FHIR profile of the BOA Imaging Study resource represents a compilation of images derived from a singular imaging study (Figure 8). The resource contains essential elements, including the unique DICOM identifier (“identifier: DICOM”) and the standard elements such as “started,” “numberOfSeries,” and “series.”

## Discussion

### Principal Results

The study presents 4 FHIR profiles that facilitate the interoperable usage of image-based biomarkers acquired by AI segmentation models, building a connection among image studies, series, and AI-derived measurements. The implementation of these FHIR profiles, as presented in this study, signifies an advancement in pursuing the clinical and interoperable integration of opportunistic screening. The usage of CT-derived body composition biomarkers in an interoperable health care ecosystem through these profiles can potentially improve clinical practice and research.

Unlike previous research [40] on enhancing the usability of existing data (eg, extracting information from reports), this study concentrated on integrating body composition data and organ measurements delivered by image-based AI models. Body composition analysis and organ measurements are important in advancing the field of personalized medicine beyond traditional measures like BMI [41,42]. For example, a high BMI does not always correlate with an increased risk of metabolic disease, but factors like sarcopenia can be associated with metabolic disease in people with normal BMI [43]. Numerous studies investigated the connection between these parameters and pathology, such as cardiovascular disease [44], diabetes [45], cancer [46,47], and metabolic disease [48,49]. With the development of AI models, many studies (more than 350 search results from databases like Web of Science [43]) have used AI techniques to assess body composition.

The standardization of body composition data and organ measurements into FHIR profiles might have significant implications for clinical practice and research. For example, by taking body composition parameters into account, clinicians could monitor the state and progression of the disease more effectively (eg, sarcopenia, a feature of chronic protein malnutrition in cirrhosis [50]) and evaluate treatment response by quantifying the changes in muscle and fat composition [47]. Presenting this information in a standardized format ensures consistency and reliability across different health care settings, facilitating more informed and timely clinical decisions. Furthermore, the interoperability of FHIR profiles allows for seamless data exchange between disparate health systems [51,52], reducing errors and enhancing the overall quality of patient care. The benefits of adopting FHIR profiles extend beyond the clinical domain to encompass research and public health. The use of standardized data formats promotes efficient data sharing among researchers, thereby fostering collaboration and accelerating the pace of scientific discovery. The accessibility and interpretability of body composition data afforded by these profiles can facilitate a multitude of research initiatives spanning clinical trials and population health studies. Furthermore, the flexibility of the FHIR framework allows for customization, facilitating the development of innovative solutions and accommodating diverse study designs.

The advent of AI in health care has introduced novel dimensions to the utility of FHIR profiles. AI applications can act as both consumers and producers of data. As consumers, AI algorithms can access standardized body composition data and organ measurements through FHIR profiles, enabling more precise and efficient analysis or predictions. These applications (eg, [47]) can enhance diagnostic accuracy, predictive analytics, and personalized treatment recommendations by leveraging interoperable data sources. Conversely, as a result of their data-generating capabilities, AI applications produce substantial quantities of valuable information, including model predictions and insights derived from data analysis. In order to fully leverage the potential of AI-generated data, it is essential to integrate it into the health care ecosystem in a standardized and interoperable format, such as FHIR, backed by Implementation Guides [53], to ensure profile conformance. This conformance can improve data quality, which builds the foundation for decision-making in health care delivery and secondary use in the research context. Such FHIR profiles ensure that insights generated by AI are accessible, interpretable, and usable across a range of health care platforms and systems. The creation of interoperable profiles for AI model predictions will facilitate the continuous improvement of AI algorithms and their integration into clinical workflows, thereby enhancing the overall quality and efficiency of patient care.

### Provenance of AI-Generated Data

FHIR profiles define how data are exchanged across different health care systems. When AI-generated data are introduced in FHIR as shown in this study, it is essential for the receiver to know the data is produced by AI models. Without identification, particularly in those health care platforms where human and AI-generated data coexist, the interpretation of clinical data can be prone to error. To help understand the context of the data, the HL7 Workgroup has introduced a Provenance Tag called “AIAST” (Artificial Intelligence Asserted), which is defined by FHIR [54] to mark the data asserted by AI models, which appears in resources’ metadata field [55]. Tagging data derived by AI models allows clinicians to trace it back to its source, reducing the risk of misinterpretation, supporting informed decisions, and further ensuring data transparency and reliability in the decision-making process.

### Application

Various studies have highlighted the significance of body composition metrics in assessing health status and predicting clinical outcomes, which are relevant to multiple clinical endpoints and diseases. Adopting FHIR profiles tailored to these body composition data offers advantages for both real-world practice and research [31,56-59]. For example, these profiles can be used in FHIR-based dashboard applications (Figure S1 in Multimedia Appendix 2), which allows the combinations of those image-based biomarkers in different clinical settings and disciplines. Through standardized storing and retrieving of body composition data, health care providers can monitor trends over time, detect diseases early, and access treatment effectiveness more accurately. Additionally, clinicians can analyze long-term patterns to gain deeper insights into chronic diseases and their associations with body composition changes, leading to better risk stratification. Furthermore, these profiles can enhance effective collaborations in multicenter research by ensuring standardized data collection and improving data comparability across studies, which can further support large-scale studies.

One particularly promising application is opportunistic screening. To illustrate, a radiological scan conducted for an alternative purpose may yield data regarding a patient’s bone density or fat distribution and may suggest an elevated risk for conditions such as cardiovascular disease [60]. By using FHIR profiles to normalize and integrate this opportunistic screening data, health care providers can incorporate these measurements into the patient’s health record, thus enabling proactive management and monitoring.

Furthermore, given the variety of AI models available from both open-source and vendor perspectives, the presented profiles can be seen as a first step in developing more advanced applications. For example, these profiles can be used to further adapt image-based AI models for future FHIR profiles.

### Limitations

While the FHIR profiles introduced in this study demonstrate a novel approach to handling data produced by AI models, certain limitations must be acknowledged. One of the limitations is the incomplete ontologies in FHIR, particularly in the imaging domain. Since they cannot fully cover all necessary concepts, custom code systems still need to be created. Additionally, there are challenges to adoption stemming from variations in technological infrastructure and FHIR versions across institutions, which can complicate the effective use of profiles. Health care systems may also be governed by distinct regulations or have unique workflows, presenting additional challenges when aligning FHIR profiles with the specific needs of these systems. The lack of standardization across institutions further exacerbates these issues, making harmonization efforts crucial for widespread adoption.

Furthermore, the successful implementation and adoption of FHIR profiles necessitate ongoing collaboration among health care providers, researchers, and organizations. As new imaging technologies and AI algorithms emerge, the profiles must evolve to incorporate these advancements, ensuring their continued relevance and effectiveness. Another critical consideration is the need for education and training for health care professionals to facilitate the adoption and integration of FHIR profiles into health care systems. Enhancing awareness of their advantages and providing the necessary support for effective usage will allow the health care community to fully capitalize on these profiles to improve patient care and outcomes.

Profiles like the presented ones hold great promise for advancing personalized medicine, and their widespread adoption also requires addressing barriers such as variations in health care systems, differing priorities among institutions, and the ongoing need for global standardization efforts. These challenges highlight the importance of collaborative and iterative development to ensure that the profiles can meet the diverse needs of the global health care community.

### Future Implementations

Given the ongoing development of FHIR, there are alternative approaches to implementing profiles to enhance their interoperability.

Imaging Selection, first introduced in FHIR Release 5, provides an efficient way to reference specific sets of images. It can be seen as a lightweight alternative to Imaging Study, enabling fast access to imaging data. Imaging Selection proves a more efficacious solution than Imaging Study in scenarios where only specific images need to be shared, as it can help minimize data size when sharing between different systems. Therefore, Imaging Study can be replaced by Imaging Selection in the future implementation of BOA profiles.

Another alternative idea for future implementation is to involve the resource Body structure, which defines a standard representation of the anatomical structure, such as body parts and organs, capturing important anatomical attributes. This resource can map the body structure found in BOA Body Structure Volume Observation in coded form. By referencing Body structure, the Observation resource will not require adjustment when additional anatomical structures are introduced but remains open to extensions, aligning with the Open Closed Principle. Furthermore, this explicit relationship simplifies query processes, enabling developers to easily retrieve all observations related to specific body structures (eg, this could allow for queries like “all Observations for the spleen”) without needing to know which components to include in the search.

### Conclusions

The BOA profiles introduced in this study provide a standardized framework for integrating complex biomarkers and organ measurements using AI algorithms in FHIR, thereby improving data interoperability and accessibility in health care. This approach seeks to improve clinical practice and patient outcomes, as well as drive innovation in research. As a first step toward sustainable and interoperable BOA model predictions, continued collaboration and adaptation will be essential to realize its full potential. The profiles serve as a foundation for future advances in image-based biomarker and organ measurement profiling.

## Appendix

Multimedia Appendix 1 Examples of value sets.

Multimedia Appendix 2 A prototype dashboard use case by Siemens Healthineers.

## Abbreviations

AI: artificial intelligence
AIAST: artificial intelligence asserted
BCA: Body Composition Analysis
BOA: Body and Organ Analysis
CT: computed tomography
DICOM: Digital Imaging and Communications in Medicine
FHIR: Fast Healthcare Interoperability Resources
FSH: FHIR shorthand
HL7: Health Level Seven
MII: Medical Informatics Initiative
MRI: magnetic resonance imaging
SNOMED CT: Systematized Nomenclature of Medicine Clinical Terms

## Data Availability

The data generated or analyzed during this study are available in the University Medicine Essen GitHub repository [61].
