# Supplementary material for: Exchange of Quantitative Computed Tomography Assessed Body Composition Data Using Fast Healthcare Interoperability Resources as a Necessary Step Toward Interoperable Integration of Opportunistic Screening Into Clinical Practice: Methodological Development Study
Source: J Med Internet Res. 2025 May 21;27:e68750. doi: 10.2196/68750 (PMC12138298; doi:10.2196/68750)
Supplement: Multimedia Appendix 1 [file jmir_v27i1e68750_app1.docx]

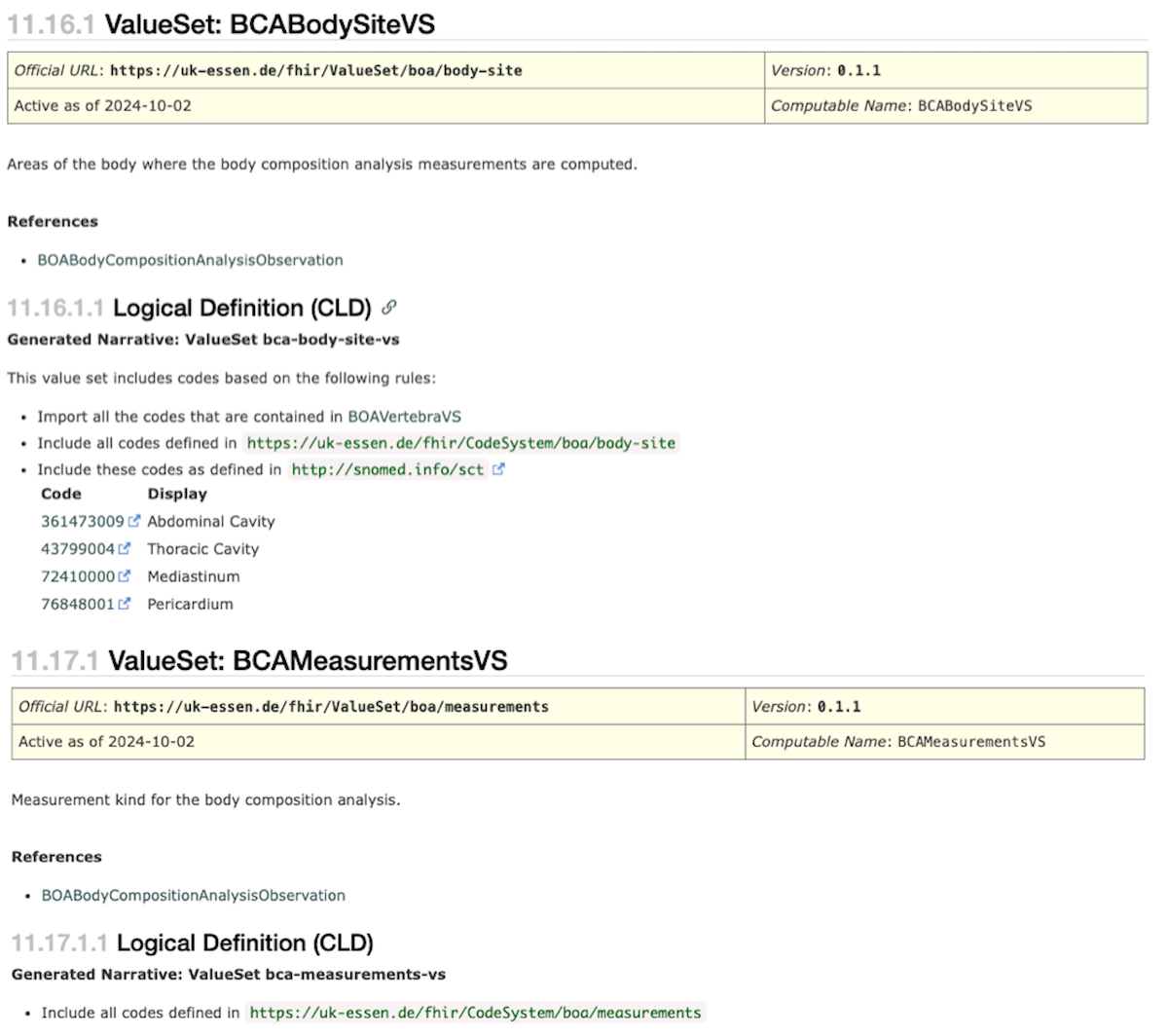


**Figure S2.** An overview of the “BCAMeasurementsVS” and “BCABodySiteVS”. The “Code” in the figure is defined in SNOMED CT (Systematized Nomenclature of Medicine – Clinical Terms) and “bodySite” (ventral cavity or whole scan). The “Display” provides the corresponding term in plain language.
