# Supplementary material for: Exchange of Quantitative Computed Tomography Assessed Body Composition Data Using Fast Healthcare Interoperability Resources as a Necessary Step Toward Interoperable Integration of Opportunistic Screening Into Clinical Practice: Methodological Development Study
Source: J Med Internet Res. 2025 May 21;27:e68750. doi: 10.2196/68750 (PMC12138298; doi:10.2196/68750)
Supplement: Multimedia Appendix 2 [file jmir_v27i1e68750_app2.docx]

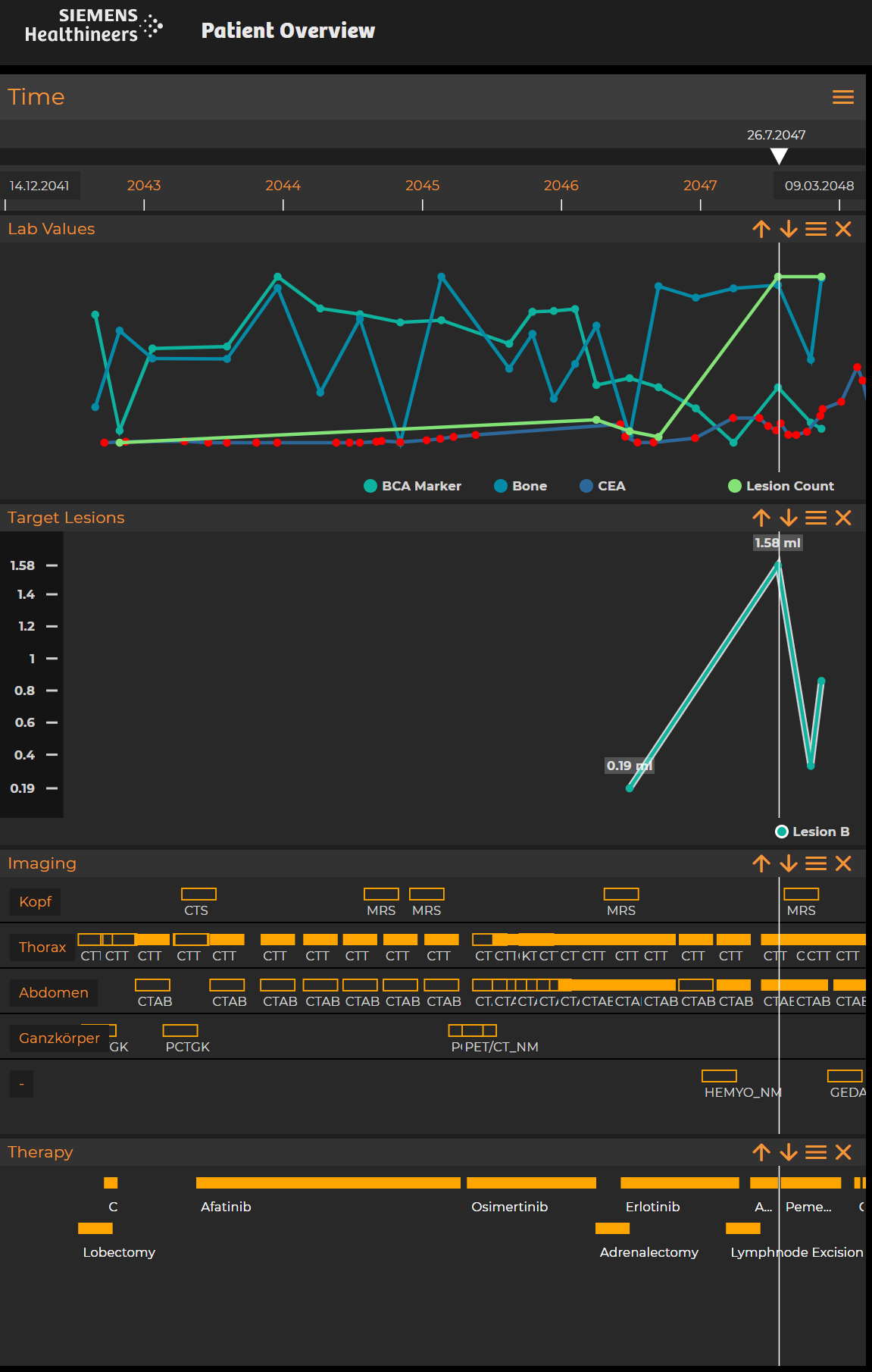


**Figure S1.** Visualization of an example use case demonstrating the integration of FHIR profiles within a prototype dashboard developed by Siemens Healthineers. This prototype illustrates how structured imaging biomarkers, such as body composition analysis (BCA) features, can be incorporated into clinical dashboards to support longitudinal patient monitoring. The dashboard displays a timeline of radiological imaging in conjunction with clinical parameters and therapy data for an oncological patient. Quantitative BCA features—including bone volume and a derived composite marker based on muscle and bone metrics—are extracted from multiple CT scans and visualized alongside clinical tumor markers (eg, CEA) and lesion counts. This example highlights the potential utility of combining imaging-derived and clinical data to enhance the visualization of disease progression and therapy monitoring.
